# Age and Cultural Differences in Recognitions of Emotions from Masked Faces among Koreans and Americans

**DOI:** 10.3390/ijerph181910555

**Published:** 2021-10-08

**Authors:** Jungsuk Kang, Seonwoo Kang, Eunbyeol Jeong, Eun-Ho Kim

**Affiliations:** Department of Psychology, Jeonbuk National University, Jeonju-si 54896, Jeollabuk-do, Korea; jungsuk.kang@jbnu.ac.kr (J.K.); parrot@jbnu.ac.kr (S.K.); starvin37@naver.com (E.J.)

**Keywords:** facial expressions, emotions, wearing masks, age difference, cultural difference

## Abstract

This study investigates age and cultural differences in the negative effects of senders’ wearing masks on receivers’ readabilities of senders’ facially expressed emotions in interpersonal interactions. An online experiment was thus conducted with Koreans and Americans aged over 20 years. Based on sampling quotas by nationality, age group and gender, Korean (*n* = 240) and American (*n* = 273) participants were recruited from panel members of a Korean research company and Amazon’s Mechanical Turk via email and the website, respectively. The participants played receiver roles to infer senders’ facially expressed emotions presented in photos in the experiment. They judged emotions facially expressed by the senders without masks and with masks are shown in photos. The results revealed that the senders’ wearing masks reduced the readabilities of the senders’ facially expressed anger among participants aged 30–49 years more than among participants aged 20–29 years. The senders’ wearing masks decreased the readabilities of the senders’ facially expressed fear for participants in their 50’s more than for participants in their 20’s. When the senders wore masks, the readabilities of the senders’ facially expressed happiness dropped among participants aged over 60 years more than among participants aged 20–49 years. When senders wore masks, American participants’ readabilities of disgust, fear, sadness and happiness expressed in the senders’ faces declined more than Korean participants’ readabilities of those emotions. The implications and limitations of these findings are discussed.

## 1. Introduction

Facial expressions of emotions serve as externally perceivable signs that are communicated between individuals in a variety of interpersonal interactions [1]. For individuals displaying facial emotional expressions (i.e., senders), their facial emotional expressions provide others (i.e., receivers) with information about their emotional states and behavioral intentions [2,3,4]. Such information elicits and reinforces the receivers’ emotional and behavioral responses expected by the senders [5]. On the other hand, the receivers automatically and quickly recognize the senders’ emotions from the senders’ facial expressions. The emotions encourage the receivers to infer the senders’ thoughts and the situational cues for their socially appropriate actions [6,7,8,9]. Hence, the exchanges of the emotions expressed in the senders’ faces between the senders and the receivers can facilitate interpersonal interactions between them [5,10,11], which result from a coevolution of display and attunement rules associated with facial emotional expressions throughout human history [1]. To form and fortify stable and positive interpersonal relationships, it is crucial for the senders to accurately convey their emotions to the receivers via their facial expressions and for the receivers to precisely read the emotions from the senders’ facial expressions in social interactions.

COVID-19 has recently spread around the world, and so people from almost all countries around the globe have favored the use of face masks in public to prevent COVID-19 infections through the airways [12,13]. Although COVID-19 vaccines are administered in many countries, these vaccines cannot effectively suppress the pandemic without mask use [14]. However, the senders’ wearing of masks may interrupt the efficient exchanges of emotions signaled in the senders’ facial expressions between the senders and the receivers during interpersonal communications [15,16]. In particular, the senders have a difficulty conveying their emotions to the receivers via their faces, partially occluded by masks in the communications. The receivers also cannot read the senders’ accurate emotions from the senders’ faces as the facial expressions of these emotions are partially covered by masks. Consequently, the receivers are more likely to react emotionally and behaviorally, contrary to the senders’ expectancies which are presented in their facial emotional expressions. When the senders observe the receivers’ unexpected emotional and behavioral responses to their facial emotional expressions, they may experience and express negative emotions (e.g., embarrassment, depression, and anger) to the receivers [17]. The senders’ negative responses can worsen the interpersonal relationships between the senders and the receivers [18]. In such situations, the senders who are wearing masks can exaggerate their facial expressions and/or use verbal and body (e.g., gestures) languages to accurately convey their emotions to the receivers [19,20]. However, the receivers have no alternative to resolve the uncertainties in recognizing the senders’ emotions from the senders’ faces partially covered by masks. When the senders are wearing masks to prevent COVID-19 infections, the receivers’ readabilities of the senders’ facially expressed emotions are of primary importance for forming and fortifying the social interactions between them.

The age-related difference in the effects of the senders’ masked faces on the receivers’ readabilities of the senders’ facially expressed emotions recently received attention from researchers [16,21]. As the receivers age, their abilities to recognize the senders’ emotions (e.g., fear, anger, sadness) accurately from the senders’ faces tend to decrease due to the age-related reduction in brain regions such as the frontal and temporal lobes, which are used for the recognition of emotions, and general cognitive declines (e.g., speed of processing) [22,23]. Hence, the senders’ wearing masks may more strongly reduce the accuracy of recognizing the senders’ emotions from the senders’ faces for older receivers than for younger receivers. Past research partly supports such possibilities. For children aged 7–13 years, the senders’ wearing masks did not reduce the accuracy of recognizing the senders’ emotions displayed in the senders’ facial expressions [21]. The receivers’ correct recognition rates of the senders’ facially expressed emotions remarkably dropped among adults aged 18–87 years when the senders wore masks [16]. According to this study’s findings, the senders’ wearing masks during the COVID-19 pandemic may have more negative effects on the accuracy of recognizing the senders’ facially expressed emotions for older receivers than for younger receivers. However, the age-related difference in the negative effects of the senders’ masked faces on the readabilities of senders’ facially expressed emotions among receivers aged over 20 years was not empirically investigated. The following research question is thus formulated and tested in this study.

Research Question 1. Is there a difference in the receivers’ accuracies to recognize the senders’ facially expressed emotions partially occluded by masks that the senders are wearing across receiver age groups ranging from those aged 20 years to those over 60 years old?

The cultural difference in the receivers’ readabilities of the senders’ facially expressed emotions was investigated by many researchers [24]. With regard to the receivers’ recognition of the accuracies of emotions shown in the senders’ masked faces, it is worth noting that the masks cover the lower part of the face (i.e., mouth), allowing the upper part of the face (i.e., eyes) to remain visible in interpersonal communications between the senders and the receivers. Recent studies showed that there was a difference in the face detection strategies for the receivers to infer the senders’ emotions from the senders’ facial expressions between Eastern and Western cultures. Eastern cultures insist on individuals’ relatedness and harmony with others (i.e., collectivist cultures) while the norm of Western cultures emphasizes that individuals become independent and unique from others (i.e., individualist cultures) [25]. Hence, Easterners are motivated to hold the interdependent construal of the self and find a way to fit in with others. Unlike Easterners, Westerners tend to hold the independent construal of the self and attend primarily to the inner states of the self [26,27]. To make emotional and behavioral responses socially acceptable, the receivers of Eastern cultures will try to infer the senders’ genuine emotions from the senders’ facial expressions more than the receivers of Western cultures. The Eastern receivers tend to focus on the senders’ eyes to accurately infer the senders’ emotional states because the eyes may reflect the senders’ genuine emotions more than their mouths [3,11,28]. In addition, the negative emotions (e.g., anger, sadness) that can threaten the harmonious relationships between the senders and the receivers are primarily recognized from the eyes [29,30,31,32]. For these reasons, the Eastern receivers who are concerned about interpersonal harmony and cooperation may attend to the senders’ eyes by default.

As discussed above, the Eastern receivers (e.g., Japanese, Chinese) are more likely to recognize the senders’ emotions displayed in the senders’ facial expressions from the senders’ eyes, while the Western receivers (e.g., British, American) tend to attend to the senders’ mouths to recognize the senders’ emotions conveyed by the senders’ facial expressions [33,34,35,36]. Consequently, the senders’ wearing masks during the COVID-19 pandemic may reduce the receivers’ readabilities of the senders’ facially expressed emotions for the Western receivers of individualist cultures more than for the Eastern receivers of collectivist cultures. However, a previous study revealed that, for Western receivers, some emotions facially expressed by the senders (e.g., sadness) are detected from the senders’ eyes whereas others (e.g., happiness) are recognized from senders’ mouths [29]. The senders’ wearing of masks will not reduce the readabilities of specific emotions facially expressed by senders (e.g., sadness) among Western receivers because the face masks cover senders’ mouths, but not eyes. Hence, there may be no cultural difference in the negative effects of the senders’ masked faces on the readabilities of the specific facial emotions displayed by the senders (e.g., sadness) between Eastern and Western receivers. The following research question is posited and investigated in this study since there is no empirical evidence addressing which types of emotions facially expressed by the senders with masks are easy or difficult to recognize for Western receivers.

Research Question 2. Is there a cultural difference in the negative effects of the senders’ wearing of masks, which occlude their mouths, on the accuracies to recognize the senders’ emotions from the senders’ faces between the Eastern receivers and the Western receivers?

To investigate these two research questions, an online experiment was conducted with Korean (Easterners of a collectivist culture) and American (Westerners of an individualist culture) participants. Research Question 2 is based on the assumption that collectivism is stronger for Korean participants than for American participants [25,37]. Hence, this study measures collectivism among all the participants in the online experiment and explores the difference in the collectivism between Korean and American participants.

## 2. Methods

### 2.1. Participants

The study protocol was approved by the institutional review board of a large national university in Korea. Compared to people living in other countries, Koreans held a stronger collectivistic view whereas Americans were more individualistic [25,37]. Hence, Koreans and Americans aged over 20 years were selected as study populations of collectivist and individualist cultures, respectively.

Based on 20 sampling quotas by nationality, age group and gender (see Table 1), Korean and American participants were recruited from panel members of a Korean research company and Amazon’s Mechanical Turk via email and the website, respectively. Each sampling quota included at least 10 participants. Two hundred and forty Koreans (mean age = 39.28, *SD* =13.44; females: 50%) and 273 Americans (mean age = 42.10, *SD* =14.87; females: 47.25%) participated in an online experiment (see Table 1). A screening question confirmed that all participants had worn face masks during the COVID-19 pandemic. All participants consented to voluntarily participate in the online experiment. Participants played receiver roles to infer senders’ facially expressed emotions presented in photos in the online experiment. A power analysis targeting a repeated measured ANOVA with 2 (participant nationalities: Koreans vs. Americans) × 5 (participant age groups ranging from 20-year-olds to over 60-year-olds) × 2 (sender races: Asians vs. Caucasians) × 2 (sender gender: males vs. females) × 6 (sender emotions: anger, disgust, fear, sadness, happiness, and surprise) × 2 (sender face coverings: no mask vs. mask) mixed design was conducted. (a) Participants’ nationalities and age groups were between-participants factors and (b) senders’ genders, emotions and face coverings were within-participants factors. The results confirmed that the sample size of the online experiment (*N* = 513) was sufficient to detect a medium effect size of *f* = 0.25 [38] at the 0.05 level in the mixed design. 

### 2.2. Stimuli

All photos of the senders’ facial emotional expressions as experimental stimuli were obtained from the Matsumoto and Ekman’s Japanese and Caucasian Facial Expressions of Emotion (JACFEE) photo set [39]. The photo set included the six basic emotions that are anger, disgust, fear, sadness, happiness, and surprise, universally expressed in senders’ faces across different cultures [40,41]. Experimental stimuli were developed through the following two stages.

In the first stage, the senders’ 2 (races: Asians vs. Caucasians) × 2 (genders: males vs. females) × 6 (emotions: anger, disgust, fear, sadness, happiness, and surprise) face photos (total of 24 face photos) were selected from the JACFFE photo set for no mask conditions used in the experiment. Six male or six female Asians’ faces and six male or six female Caucasians’ faces corresponded to six emotions.

At the next stage, one image of a typical mask for both Korean and American participants was chosen after identifying the sales of masks on famous Korean (www.coupang.com, accessed on 13 May 2021) and American (www.amazon.com, accessed on 13 May 2021) internet shopping sites among Koreans and Americans, respectively. To develop the photo set presented by the mask conditions, the mask image was visually applied to the face photos of Asians and Caucasians selected in the previous stage by using Photoshop. The masks covered approximately 40% of Asians’ and Caucasians’ faces shown in the photos. 

Finally, the senders’ 2 (races: Asians vs. Caucasians) × 2 (genders: males vs. females) × 6 (emotions: anger, disgust, fear, sadness, happiness, and surprise) × 2 (face coverings: no mask vs. mask) face photos (totally 48 face photos) were developed as the experimental stimuli. The set of 24 photos of Asians’ faces and the set of 24 photos of Caucasians’ faces were randomly presented to Korean and American participants in the experiment, respectively. Consequently, each Korean (vs. American) participant was randomly exposed to (a) 6 face photos of 6 emotions facially expressed by Asian (vs. Caucasian) males without masks, (b) 6 face photos of 6 emotions facially expressed by the same males with masks, (c) 6 face photos of 6 emotions facially expressed by Asian (vs. Caucasian) females without masks, and (d) 6 face photos of 6 emotions facially expressed by the same females with masks. The Asians and the Caucasians shown in the photos were aged between 20 to 29 years.

### 2.3. Procedure

Two hundred forty Koreans and 273 Americans who were screened for their experience of wearing masks during the COVID-19 pandemic in advance completed the experiment online. All participants wore masks at least once during the COVID-19 pandemic. Informed consents were obtained online from each participant. All data were anonymously collected from all participants.

In the online experiment, Korean and American participants viewed the photos of 24 face photos of Asians and 24 face photos of Caucasians, respectively. The photos were randomly presented to participants one after another. Next, participants were asked to identify the emotion depicted on the face photo that they were viewing by selecting one of the six emotion labels (anger, disgust, fear, sadness, happiness, and surprise). They viewed each face photo and selected a specific emotion that they identified from the face photo at their own pace by using navigation links (next buttons). After completing the facial emotion recognition tasks, Korean and American participants were instructed to assess their collectivism on a 16-item, 7-point Likert-type scale (e.g., ‘It is my duty to take care of my family even when I have to sacrifice what I want,’ and ‘It is important to me that I respect the decisions made by my groups.’) adopted from a Korean study [42] (Cronbach’s alpha = 0.82) or an American study [43] (Cronbach’s alpha = 0.78), respectively. The items used to measure collectivism were semantically identical for Korean and American participants [42]. The items were developed from the following definition. Collectivism is defined as a cultural orientation for an individual to vary in the extent to which he/she emphasizes interdependent aspects of the self and communal relationships, pursues in-group goals and acts according to social norms [43].

## 3. Results

### 3.1. Checking Assumption

This study compared the collectivist tendency of Korean participants with that of American participants. An independent sample *t* test revealed that Korean participants showed a stronger collectivist tendency than American participants (*M*_Korean_ = 3.01, *M*_American_ = 2.27, *t*(510.58) = 8.59, variance equality was violated, *p* < 0.001). Therefore, the results confirmed that the Korean participants, as Easterners of a collectivist culture, had stronger collectivism than the American participants, as Westerners of an individualist culture.

### 3.2. Investigating Research Questions

Before investigating the two research questions, an index reflecting the difference in the participants’ accuracies to recognize emotions from the photos of either Asian faces (for Korean participants) or Caucasian faces (for American participants) between no mask and mask conditions were calculated for each emotion and each participant. The index (hereafter accuracy difference index) was obtained by subtracting the mean of the correct recognitions of a specific facial emotion displayed in the photos with mask conditions from the mean of the correct recognitions of the facial emotions displayed in the photos with no mask conditions for each participant. As the accuracy difference indexes increase, the accuracies to recognize facial emotions displayed in the photos with masks become lower than the accuracies to recognize the facial emotions displayed in the photos without masks.

#### 3.2.1. Comparisons of Participants’ Accuracies to Recognize the Senders’ Facially Expressed Emotions between No Mask and Mask Conditions for Participant Age Groups

Thirty paired sample *t* tests were performed to investigate the differences in the participants' accuracies to recognize senders’ facially expressed emotions between no mask and mask conditions for five participant age groups ranging from 20-year-old to over 60-year-old participants. See Table 2 for details.

First, for participants in their 20s, the accuracies to recognize disgust, fear, sadness, happiness and surprise expressed in senders’ faces were higher in no mask conditions than in mask conditions. Second, the accuracies to recognize all emotions (anger, disgust, fear, sadness, happiness, and surprise) displayed in the senders’ faces were higher in no mask conditions than in mask conditions among participants aged in their 30s. Third, for participants aged in their 40s, the accuracies to recognize anger, disgust, fear, sadness and happiness expressed in the senders’ faces were higher in no mask conditions than in mask conditions. Fourth, the accuracies to recognize disgust, fear, sadness, happiness, and surprise displayed in the senders’ faces were higher in no mask conditions than in mask conditions among participants aged in their 50s. Lastly, for the participants aged over 60 years, the accuracies to recognize disgust, fear, sadness, and happiness expressed in senders’ faces were higher in no mask conditions than in mask conditions.

#### 3.2.2. Comparisons of the Participants’ Accuracy Difference Indexes across Participant Age Groups (Research Question 1)

**Figure 1:**
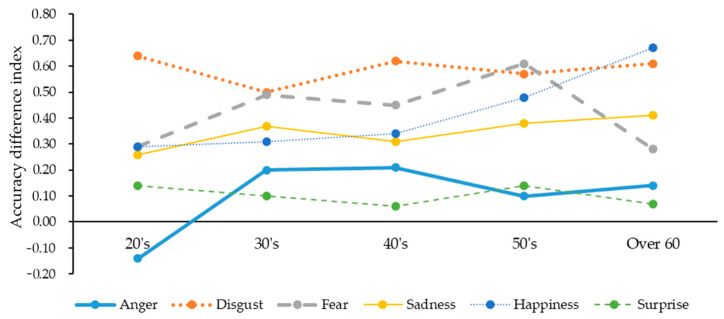
Graphs for participants’ age-related difference in accuracy difference indexes.

To explore the difference in the accuracy difference indexes across five participant age groups ranging from 20-year-old to over 60-year-old participants (Research Question 1), six one-way ANOVAs were conducted (see Table 3 and Figure 1).

First, for the anger expressed in the senders’ faces, the accuracy difference indexes differed across the participant age groups (*F*(4, 508) = 4.22, *p* < 0.005). Scheffe’s post hoc tests showed that the accuracy difference indexes for participants aged in their 30s (*M* = 0.20) and 40s (*M* = 0.21) were higher than the accuracy difference index for participants in their 20s (*M* = −0.14) (two *p*’s < 0.05). The results indicated that the senders’ wearing masks experienced a greater decrease in the accuracies to recognize senders’ facially expressed anger for participants in their 30s and 40s than for participants in their 20s. Although the accuracy difference index was found to be negative (−0.14) for participants in their 20s (see Table 3), there was no significant difference in their accuracies to recognize anger displayed in the senders’ faces between no mask and mask conditions (*t(141)* = 1.92, *p* = 0.05) (see Table 2).

Second, for the fear displayed in the senders’ faces, the accuracy difference indexes were different across the participant age groups (*F*(4, 508) = 3.57, *p* < 0.01). A Scheffe’s post hoc test revealed that the accuracy difference index for participants in their 50s (*M* = 0.61) was higher than that for participants in their 20s (*M* = 0.29) (*p* < 0.05). The results showed that the senders’ wearing masks experienced a greater decrease in the accuracies to recognize the senders’ facially expressed fear for participants in their 50s than for participants in their 20s.

Lastly, for the happiness expressed in senders’ faces, the accuracy difference indexes differed across participant age groups (*F*(4, 508) = 6.01, *p* < 0.001). Scheffe’s post hoc tests showed that the accuracy difference index for participants over 60 years (*M* = 0.67) was higher than the accuracy difference indexes for participants in their 20s (*M* = 0.29), 30s (*M* = 0.31) and 40s (*M* = 0.34) (three *p*’s < 0.005). The results indicated that senders’ wearing masks experienced a greater decrease in the accuracies to recognize the senders’ facially expressed happiness for participants in their 60s than for participants in their 20s, 30s and 40s.

#### 3.2.3. Comparisons of the Participants’ Accuracies to Recognize the Senders’ Facially Expressed Emotions between No Mask and Mask Conditions for Participant Nationality Groups

Twelve paired sample *t* tests were conducted to investigate the differences in the participants' accuracies to recognize the senders’ facially expressed emotions between no mask and mask conditions for Korean and American participants. See Table 4 for details.

For Korean participants, the accuracies to recognize all emotions (anger, disgust, fear, sadness, happiness, and surprise) expressed in senders’ faces were higher in no mask conditions than in mask conditions. For American participants, the accuracies to recognize disgust, fear, sadness, happiness and surprise were higher in no mask conditions than in mask conditions.

#### 3.2.4. Comparisons of the Participants’ Accuracy Difference Indexes between Participant Nationality Groups (Research Question 2)

**Figure 2:**
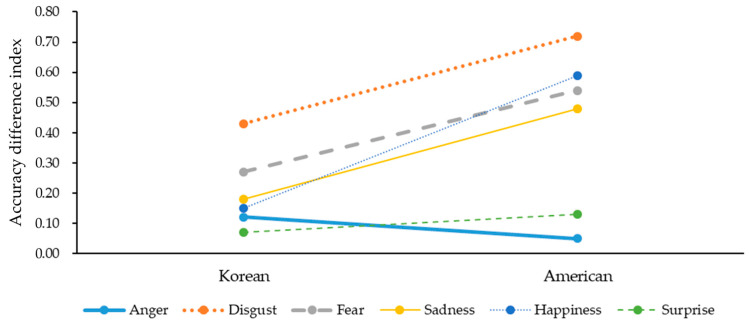
Graphs for participants’ nationality-related difference in accuracy difference indexes.

To investigate the differences in the accuracy difference indexes between Korean and American participants (Research Question 2), six independent sample *t* tests were conducted (see Table 5 and Figure 2).

First, for the disgust expressed in the senders’ faces, the accuracy difference index for American participants (*M* = 0.72) was higher than that for Korean participants (*M* = 0.43) (*t*(511) = −3.99, *p* < 0.001). Second, for the fear displayed in the senders’ faces, the accuracy difference index for American participants (*M* = 0.54) was higher than that for Korean participants (*M* = 0.27) (*t*(503.17) = −4.38, variance equality was violated, *p* < 0.001). Third, for the sadness expressed in the senders’ faces, the accuracy difference index for American participants (*M* = 0.48) was higher than that for Korean participants (*M* = 0.18) (*t*(511) = −4.50, *p* < 0.001). Lastly, for the happiness displayed in senders’ faces, the accuracy difference index for American participants (*M* = 0.59) was higher than that for Korean participants (*M* = 0.15) (*t*(491.68) = −9.32, variance equality was violated, *p* < 0.001). These results indicated that the senders’ wearing masks experienced a greater decrease in the accuracies to recognize the senders’ facially expressed disgust, fear, sadness and happiness for American participants than for Korean participants. 

## 4. Discussion

This study investigated how the senders’ wearing of masks could have differential negative effects on the receivers’ accuracies to recognize six basic emotions (anger, disgust, fear, sadness, happiness, and surprise) expressed in the senders’ faces across the receivers’ age groups (20s, 30s, 40s, 50s and over 60) (Research Question 1) and between the receivers’ nationality groups (Koreans and Americans) (Research Question 2) in interpersonal communications.

Research Question 1 was proposed to ascertain how the senders’ wearing of masks could differently decrease the receivers’ recognition accuracies of the senders’ facially expressed emotions across the receivers’ age groups. The study results revealed that, as the receivers’ ages increased, the negative effects of the senders’ wearing masks on the receivers’ recognition accuracies of the anger expressed in the senders’ faces increased and then decreased. Such an inverted-U-shaped relationship is shown in Figure 1. In particular, the senders’ wearing of masks reduced the recognition accuracies of the senders’ facially expressed anger for the receivers aged 30–49 years more than those for the receivers aged 20–29 years. Like anger, Figure 1 showed that as the receivers’ ages increased, the negative effects of senders’ wearing masks on the receivers’ readabilities of fear displayed in the senders’ faces increased and then decreased. Particularly, due to senders’ wearing masks, the recognition accuracies of the senders’ facially expressed fear decreased for receivers in their 50s more than for receivers in their 20s. In addition, as the receivers’ ages increased, the negative effects of senders’ wearing masks on the receivers’ recognition accuracies of happiness facially expressed by the senders increased. Such a relationship is depicted in Figure 1. In particular, when the senders were wearing masks, the recognition accuracies of the senders’ facially expressed happiness for the receivers over 60 years decreased more than those for the receivers aged 20–49 years.

In summary, the senders’ wearing masks reduced the recognition accuracies of the senders’ facially expressed anger, fear and happiness for older adult receivers more than those for younger adult receivers. These results were similar to past research [16,21].

Research Question 2 explored how the senders’ wearing masks could differently reduce the receivers’ recognition accuracies of the senders’ facially expressed emotions between Korean and American receivers. The study findings revealed that when senders were wearing masks, the recognition accuracies of the disgust, fear, sadness, and happiness expressed in the senders’ faces for American receivers decreased more than those for Korean receivers. These results indicated that the Korean receivers’ readabilities of the senders’ facially expressed emotions were relatively less irritated by the senders’ wearing of masks than the American receivers’ readabilities. This is because Americans, as Westerners (individualists), tend to make their emotional judgments based on the detection of senders’ mouths that are covered by masks. However, Koreans as Easterners (collectivists) are more likely to recognize senders’ facially expressed emotions from the senders’ eyes that are not occluded by masks [33,34,35,36].

When interpreting the current findings, several limitations should be considered. The study did not find the reason why age-related (anger, fear, happiness) and cultural (disgust, fear, sadness, happiness) differences in the receivers’ readabilities occurred only for some emotions. For example, emotions can be classified based on valence (positive vs. negative) and arousal (high vs. low) dimensions [44,45]. In the positive dimension, surprise is a high arousal emotion and happiness is a low arousal emotion. In the negative dimension, anger and fear are high arousal emotions and disgust and sadness are low arousal emotions. With regard to this classification, younger and older adult receivers can recognize high arousal emotions more easily than low arousal emotions. Additionally, younger adult receivers attend more to positive emotions than negative emotions whereas older adult receivers quickly detect both positive and negative emotions [46]. However, the study results cannot be interpreted by such past studies. Further studies will thus attempt to give reasons why the age differences in receivers’ readabilities happened for only some emotions.

The reason why a difference between Eastern and Western cultures in face detection strategies for the receivers to recognize the senders’ emotions from the senders’ facial expressions (focusing on eyes vs. mouths) occurs has not yet been investigated. Based on past studies [26,29,30,31,35], the authors inferred that distinct cultural orientations of collectivism and individualism may result in different face detection strategies to focus on the eyes and mouths, respectively. Hence, further studies empirically investigating the authors’ inferences are needed.

Past studies showed that the receivers could not accurately read the senders’ emotions from the senders’ facial expressions for the following reasons. First, the receivers could not read the senders’ facially expressed emotions correctly without knowing the social contexts which the senders encountered (e.g., the levels of the senders’ social needs) [47]. Second, the static view of the senders’ facial expressions could reduce the receivers’ accuracies to recognize the senders’ emotions from the senders’ faces [48]. Third, the senders’ facially expressed emotions were commonly conveyed to the receivers with other verbal and bodily (e.g., gestures) information in actual social interactions [19,20]. However, this study did not inform the receivers in interpersonal communications about the senders’ social situations. Additionally, the static photos of the senders’ facially expressed emotions without verbal and bodily information were used as experimental stimuli in this study. Further studies on the effects of senders’ wearing masks in interpersonal communications need to provide participants with situational, verbal and bodily information associated with the senders. These study findings are expected to extend our understanding of the effect of senders’ wearing masks on nonverbal communications between the senders and the receivers.

The correct judgment of emotional facial expressions can depend on the age congruence between the sender who makes his/her emotional faces and the receiver who reads those faces [49]. When the age of the sender is the same as that of the receiver, the receiver can accurately judge the emotions that the sender makes via facial expressions. It is called own-age advantage in face processing. However, the photos of younger adult senders’ emotional facial expressions were used as experimental stimuli in the current study. For this reason, future research should investigate how own-age advantage can moderate the effects of the senders’ wearing masks on the receivers’ accuracies to detect emotions from the senders’ facial expressions.

## 5. Conclusions

Face mask usage is regarded as an effective strategy to control COVID-19 transmission around the world. However, the senders’ wearing of masks can interrupt the efficient exchanges of the senders’ facially expressed emotions between the senders and the receivers during their interpersonal communications. This study’s findings suggest that the senders’ wearing masks will more greatly reduce the receivers’ recognition accuracies of some emotions (e.g., anger, fear, and happiness) facially expressed by the senders for older adult receivers than for younger adult receivers. Hence, when the senders are wearing masks to prevent COVID-19 infections, older adult receivers’ readability of senders’ facially expressed emotions can decrease and then the senders’ wearing masks may impede the social interactions between the senders and the receivers. As the COVID-19 pandemic is globally prolonged, face mask usage across the world becomes long term. It is apprehended that such long-term use of masks can lead to a progressive decline in older adults’ abilities to recognize emotions facially expressed by others in interpersonal communications [50]. Governments and researchers around the world need to develop educational programs to cope with the decline in older adults’ readabilities of emotions. This study’s findings suggest that adults aged 30–59 years need educational programs to increase their readabilities of negative emotions (e.g., anger and fear) and adults aged over 60 years need educational programs to enhance their readabilities of positive emotions (e.g., happiness). In addition, the study results propose that the American receivers’ readabilities of the senders’ facially expressed emotions (e.g., disgust, sadness, and happiness) will be irritated by the senders’ wearing masks more than the Korean receivers’ readabilities. This is because American receivers of individualist cultures tend to detect the senders’ facially expressed emotions from the senders’ mouths covered by masks. However, Korean receivers of collectivist cultures are more likely to focus on the senders’ eyes which are not occluded by masks in judging the senders’ facially expressed emotions. Consequently, the senders’ wearing masks can strongly confuse the American receivers in reading the senders’ facially expressed emotions when they communicate in social interactions. It is necessary for American governments and non-profit organizations (e.g., Centers for Disease Control and Prevention) to inform Americans that (a) matching between emotions conveyed through the face and voice, (b) the exaggeration of facial expressions and (c) the use of bodily expressions (e.g., gestures) can help to facilitate the efficient exchanges of emotions signaled in facial expressions from interpersonal situations where people are wearing masks [20,22,51]. It is also publicized that Americans who work at or visit places where emotional communications are important (e.g., hospitals and schools) need to wear transparent masks (e.g., lip-view masks or clear face masks) to intensify emotional exchanges during interpersonal communications [52].

## Figures and Tables

**Table 1:** Sub-ample sizes and average ages for experimental groups.

Nationality	Gender	20s	30s	40s	50s	Over 60
Korean	Male	38 (25.44) ^1^	27 (33.40)	28 (45.14)	14 (55.14)	13 (64.53)
	Female	35 (24.74)	39 (34.33)	17 (46.11)	19 (55.52)	10 (64.00)
	Total	73 (25.11)	66 (33.95)	45 (45.51)	33 (55.36)	23 (64.30)
American	Male	38 (26.39)	42 (34.28)	19 (44.10)	24 (54.50)	21 (69.52)
	Female	31 (26.25)	36 (33.50)	18 (43.44)	21 (53.66)	23 (65.91)
	Total	69 (26.33)	78 (33.92)	37 (43.78)	45 (54.11)	44 (67.63)

^1^ The numbers in brackets are average ages.

**Table 2:** Comparisons of participants’ accuracies to recognize senders’ facially expressed emotions between no mask and mask conditions for participant age groups.

Emotion ^1^	20s (*n* = 142)		30s (*n* = 144)		40s (*n* = 82)		50s (*n* = 78)		Over 60 (*n* = 67)	
	No Mask	Mask	No Mask	Mask	No Mask	Mask	No Mask	Mask	No Mask	Mask
Anger (*SD*)	1.14 (0.69)	1.28 (0.76)	1.49 (0.62)	1.28 (0.67)	1.45 (0.66)	1.23 (0.70)	1.46 (0.69)	1.35 (0.70)	1.32 (0.66)	1.17 (0.69)
	*t*(141) = 1.92, *p* = 0.05		*t*(143) = −3.15, *p* < 0.005		*t*(81) = −2.48, *p* < 0.05		*t*(77) = −1.27, *p* = 0.20		*t*(66) = −1.42, *p* = 0.15	
Disgust (*SD*)	1.43 (0.66)	0.78 (0.67)	1.34 (0.68)	0.84 (0.68)	1.50 (0.59)	0.87 (0.72)	1.43 (0.67)	0.85 (0.67)	1.50 (0.63)	0.89 (0.65)
	*t*(141) = −9.53, *p* < 0.001		*t*(143) = −7.20, *p* < 0.001		*t*(81)= −6.81, *p* < 0.001		*t*(77) = −5.46, *p* < 0.001		*t*(66) = −6.28, *p* < 0.001	
Fear (*SD*)	0.67 (0.68)	0.38 (0.54)	0.85 (0.76)	0.36 (0.57)	0.80 (0.82)	0.35 (0.52)	1.12 (0.70)	0.51 (0.63)	1.13 (0.81)	0.85 (0.70)
	*t*(141) = −5.16, *p* < 0.001		*t*(143) = −8.84, *p* < 0.001		*t*(81) = −5.18, *p* < 0.001		*t*(77) = −7.69, *p* < 0.001		*t*(66) = −2.99, *p* < 0.005	
Sadness (*SD*)	1.47 (0.67)	1.20 (0.66)	1.56 (0.57)	1.18 (0.75)	1.57 (0.58)	1.25 (0.68)	1.57 (0.59)	1.19 (0.62)	1.61 (0.54)	1.19 (0.76)
	*t*(141) = −4.18, *p* < 0.001		*t*(143) = −6.10, *p* < 0.001		*t*(81) = −4.73, *p* < 0.001		*t*(77) = −3.96, *p* < 0.001		*t*(66) = −3.77, *p* < 0.001	
Happiness (*SD*)	1.87 (0.33)	1.57 (0.59)	1.93 (0.24)	1.61 (0.55)	1.95 (0.21)	1.60 (0.62)	1.93 (0.24)	1.44 (0.61)	2.00 (0.00)	1.32 (0.58)
	*t*(141) = −6.65, *p* < 0.001		*t*(143) = −7.13, *p* < 0.001		*t*(81) = −4.61, *p* < 0.001		*t*(77) = −6.52, *p* < 0.001		*t*(66) = −9.35, *p* < 0.001	
Surprise (*SD*)	1.80 (0.39)	1.66 (0.51)	1.83 (0.39)	1.72 (0.49)	1.89 (0.31)	1.82 (0.40)	1.89 (0.30)	1.75 (0.51)	1.86 (0.34)	1.79 (0.40)
	*t*(141) = −3.26, *p* < 0.005		*t*(143) = −2.26, *p* < 0.05		*t*(81) = −1.14, *p* = 0.25		*t*(77) = −2.47, *p* < 0.05		*t*(66) = −1.15, *p* = 0.25	

^1^ Dependent variable = mean of correct recognitions of senders’ facially expressed emotions.

**Table 3:** Investigation of participants’ age-related difference in accuracy difference indexes.

Emotion ^1^	20s (*n* = 142)	30s (*n* = 144)	40s (*n* = 82)	50s (*n* = 78)	Over 60 (*n* = 67)
Anger (*SD*)	−0.14 (0.87)_a_	0.20 (0.79)_b_	0.21 (0.80)_c_	0.10 (0.71)_d_	0.14 (0.85)_e_
	*F*(4, 508) = 4.22, *p* < 0.005; a < b = c, *p* < 0.05				
Disgust (*SD*)	0.64 (0.80)_a_	0.50 (0.84)_b_	0.62 (0.82)_c_	0.57 (0.93)_d_	0.61 (0.79)_e_
	*F*(4, 508) = 0.56, *p* = 0.68				
Fear (*SD*)	0.29 (0.68)_a_	0.49 (0.66)_b_	0.45 (0.78)_c_	0.61 (0.70)_d_	0.28 (0.77)_e_
	*F*(4, 508) = 3.57, *p* < 0.01; a < d, *p* < 0.05				
Sadness (*SD*)	0.26 (0.76)_a_	0.37 (0.73)_b_	0.31 (0.60)_c_	0.38 (0.85)_d_	0.41 (0.90)_e_
	*F*(4, 508) = 0.64, *p* = 0.63				
Happiness (*SD*)	0.29 (0.52)_a_	0.31 (0.53)_b_	0.34 (0.67)_c_	0.48 (0.65)_d_	0.67 (0.58)_e_
	*F*(4, 508) = 6.01, *p* < 0.001; a = b = c < e, *p* < 0.005				
Surprise (*SD*)	0.14 (0.51)_a_	0.10 (0.55)_b_	0.06 (0.48)_c_	0.14 (0.50)_d_	0.07 (0.53)_e_
	*F*(4, 508) = 0.45, *p* = 0.76				

^1^ Dependent variable = accuracy difference index. Signs of _a_, _b_, _c_, _d_ and _e_ indicate the accuracy difference indexes (dependent variable) for participants aged 20–29 years, 30–39 years, 40–49 years, 50–59 years and over 60, respectively.

**Table 4:** Comparisons of participants’ accuracies to recognize senders’ facially expressed emotions between no mask and mask conditions for participant nationality groups.

Emotion ^1^	Korean (*n* = 240)		American (*n* = 273)	
	No Mask	Mask	No Mask	Mask
Anger (*SD*)	1.21 (0.70)	1.08 (0.72)	1.49 (0.63)	1.43 (0.66)
	*t*(239) = −2.13, *p* < 0.05		*t*(272) = −1.29, *p* = 0.19	
Disgust (*SD*)	1.19 (0.69)	0.75 (0.65)	1.64 (0.54)	0.91 (0.69)
	*t*(239) = −7.80, *p* < 0.001		*t*(272) = −15.05, *p* < 0.001	
Fear (*SD*)	0.39 (0.61)	0.11 (0.32)	1.29 (0.63)	0.74 (0.64)
	*t*(239) = −7.11, *p* < 0.001		*t*(272) = −11.56, *p* < 0.001	
Sadness (*SD*)	1.30 (0.64)	1.11 (0.68)	1.76 (0.47)	1.28 (0.71)
	*t*(239) = −3.63, *p* < 0.001		*t*(272) = −10.97, *p* < 0.001	
Happiness (*SD*)	1.92 (0.26)	1.77 (0.46)	1.93 (0.24)	1.33 (0.62)
	*t*(239) = −5.15, *p* < 0.001		*t*(272) = −15.67, *p* < 0.001	
Surprise (*SD*)	1.85 (0.35)	1.77 (0.45)	1.84 (0.36)	1.71 (0.50)
	*t*(239) = −2.26, *p* < 0.05		*t*(272) = −4.38, *p* < 0.001	

^1^ Dependent variable = mean of correct recognitions of senders’ facially expressed emotions.

**Table 5:** Investigation of participants’ nationality-related difference in accuracy difference indexes.

Emotion ^1^	Korean (*n* = 240)	American (*n* = 273)
Anger (*SD*)	0.12 (0.90)	0.05 (0.74)
	*t*(463.67) ^2^ = 0.89, *p* = 0.36	
Disgust (*SD*)	0.43 (0.86)	0.72 (0.79)
	*t*(511) = −3.99, *p* < 0.001	
Fear (*SD*)	0.27 (0.60)	0.54 (0.78)
	*t*(503.17) ^2^ = −4.38, *p* < 0.001	
Sadness (*SD*)	0.18 (0.78)	0.48 (0.72)
	*t*(511) = −4.50, *p* < 0.001	
Happiness (*SD*)	0.15 (0.45)	0.59 (0.62)
	*t*(491.68) ^2^ = −9.32, *p* < 0.001	
Surprise (*SD*)	0.07 (0.51)	0.13 (0.52)
	*t*(511) = −1.39, *p* = 0.16	

^1^ Dependent variable = accuracy difference index. ^2^ Variance equality was violated.

## Data Availability

Not applicable.
